# Synergetic Near- and Far-Field Plasmonic Effects for Optimal All-Perovskite Tandem Solar Cells with Maximized Infrared Absorption

**DOI:** 10.1021/acs.jpclett.4c00194

**Published:** 2024-02-29

**Authors:** Jaime Bueno, Sol Carretero Palacios, Miguel Anaya

**Affiliations:** † Instituto de Ciencia de Materiales de Madrid, ICMM-CSIC, C/Sor Juana Inés de la Cruz, 3, 28049 Madrid, Spain; ‡ Departamento de Física de la Materia Condensada, Instituto de Ciencia de Materiales de Sevilla, Universidad de Sevilla-CSIC, Av. Reina Mercedes SN, Sevilla 41012, Spain

## Abstract

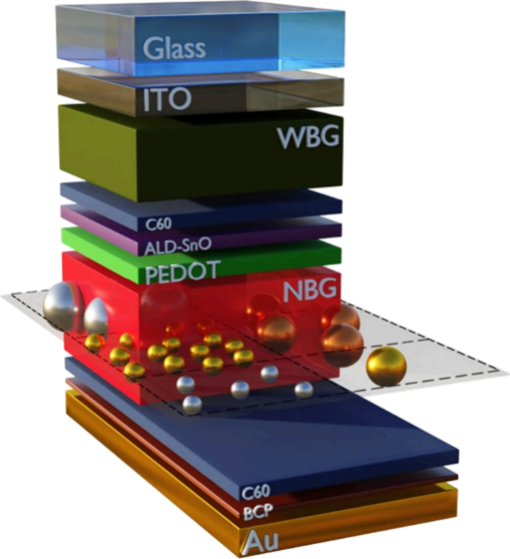

The efficiency and reliability of perovskite solar cells have rapidly increased in conjunction with the proposition of advanced single-junction and multi-junction designs that allow light harvesting to be maximized. However, Sn-based compositions required for optimized all-perovskite tandem devices have reduced absorption coefficients, as opposed to pure Pb perovskites. To overcome this, we investigate near- and far-field plasmonic effects to locally enhance the light absorption of infrared photons. Through optimization of the metal type, particle size, and volume concentration, we maximize effective light harvesting while minimizing parasitic absorption in all-perovskite tandem devices. Interestingly, incorporating 240 nm silver particles into the Pb–Sn perovskite layer with a volume concentration of 3.1% indicates an absolute power conversion efficiency enhancement of 2% in the tandem system. We present a promising avenue for experimentalists to realize ultrathin all-perovskite tandem devices with optimized charge carrier collection, diminishing the weight and the use of Pb.

Over the past decade, halide perovskites have emerged as a game changer for the demonstration of highly efficient optoelectronic devices. In particular, their efficient light absorption, low nonradiative recombination rate, and ease of processing have enabled a rapid increase in the efficiency of photovoltaic (PV) devices, surpassing 25% in single-junction solar cells.^1^ Although these performances bring them to the level of the performances of mature technologies such as silicon (Si) PVs, beyond stability issues they are fundamentally limited by thermodynamic limits, as described by the detailed balance model.^2^ In this context, multi-junction devices comprised of two or more absorbing subcells combining perovskites with Si, CIGS, and organic semiconductors have been proposed to surpass those theoretical limits by complementary absorbing different regions of the solar spectrum.^3−5^ Interestingly, a perovskite/silicon tandem device with a power conversion efficiency (PCE) of 33.2% has been demonstrated recently,^1^ exceeding the efficiency limit of a single-junction solar cell.

One of the most striking properties of halide perovskites is the tunability of their bandgap by compositional engineering. This favors the development of all-perovskite tandem devices employing bandgap combinations that maximize both the collection of solar photons and the open circuit voltages (*V*_oc_) of the tandem device. This advantage over other more commercially established semiconductors makes it possible to propose all-perovskite tandem solar cells, holding great promise for their emergence as top contenders for ultraefficient third-generation PVs.^6−8^ Indeed, monolithic perovskite/perovskite tandem devices have already surpassed their single-junction counterparts, achieving a remarkable record PCE of 28%,^9^ compared to the value of 26.1% achieved by conventional architectures.^1^ However, this value still lags behind the theoretical ceiling. While lead (Pb)-based wide bandgap (WBG) perovskites with energy bandgaps (*E*_g_) of >1.7 eV exhibit excellent light absorption, narrow bandgap (NBG) perovskites with an *E*_g_ of <1.25 eV, resulting from the inclusion of tin (Sn) in the composition at the expense of Pb,^10^ demonstrate suboptimal light harvesting properties. This hinders the ability to obtain all-perovskite tandem solar cells with efficiencies near their theoretical limits. The conventional approach for addressing this challenge involves employing thicker Pb–Sn perovskite layers to achieve full light extinction at the rear subcell. However, the practical realization of thick perovskite layers (∼1 μm) capable of enhancing light absorption while preserving efficient charge extraction is fraught with experimental difficulties. Only a limited number of research teams have successfully tackled this demanding task.^6,11^

An alternative approach for maximizing light absorption and, consequently, the short circuit current (*J*_sc_) in a solar cell is to control light–matter interactions through optical design.^12^ For example, nanophotonic structures such as gratings and scatterers have been successfully employed to increase light trapping and thus the photocurrent in perovskite solar cells.^13−15^ Similarly, pyramids imprinted in Si devices are typically used to maximize performance in perovskite/Si tandem devices.^16^ However, these are complex strategies, especially for thin film systems in which the absorption has to be enhanced selectively within the cross section of the device, as it is required to boost light harvesting in the rear subcell of a perovskite/perovskite tandem solar cell. In contrast, a highly promising and less complex route to maximize light absorption and *J*_sc_ involves the strategic use of plasmonic nanoparticles (NPs). These NPs, by leveraging both near- and far-field effects, offer a surgical means for influencing light management precisely within the spectral and spatial regions of interest.^17−20^ While plasmonic NPs have been successfully employed to enhance the absorption of MAPbI_3_,^21−23^ aimed at reducing the active layer thickness and mitigating toxicity associated with the Pb content, this innovative approach has yet to be applied to Sn-based perovskites within tandem devices. The primary challenge has been the difficulty in achieving high scattering and near-field effects in the crucial near-infrared (NIR) spectral region. Nonetheless, harnessing the potential of plasmonic NPs in this context holds great promise for advancing the performance of tandem solar cells, offering a nuanced solution to the challenges posed by traditional approaches.

Herein, we design the novel concept of a perovskite/perovskite tandem solar cell in which metallic NPs that support localized surface plasmon resonances (LSPRs) are directly embedded in the Pb–Sn perovskite layer to boost the PCE by optimizing light absorption. For this purpose, we perform finite-difference time-domain (FDTD) calculations using the commercial software Ansys Lumerical FDTD,^24^ by which the propagation of electromagnetic waves along the Pb–Sn perovskite-based single-junction and tandem solar cells are simulated. We screen a multiparametric space with plasmonic NPs of different composition, size, and concentration randomly distributed within the Pb–Sn perovskite layer to maximize light harvesting while minimizing parasitic absorption. To design an all-perovskite tandem solar cell that maximizes current matching, we begin by optimizing the combination of both near- and far-field plasmonic effects in a single-junction device. In this context, we have identified large silver (Ag) NPs with resonances located in the NIR region as the most promising candidates for this purpose. Our calculations show that when 120 nm radius Ag NPs are embedded in a 700 nm thick NBG perovskite layer of a tandem device with a volume concentration of 3.1%, the calculated PCE reaches 33.34%. This represents an absolute increase of 2% compared with that of the reference counterpart. To achieve a similar enhancement in a plasmon-free cell, one would need to double the thickness of the Pb–Sn perovskite layer. Hence, this work provides a user’s guide for experimentalists to apply solutions to obtain highly absorbing thin NBG layers that allow charge extraction, overcoming manufacturing challenges in all-perovskite tandem solar cells.

We simulate first the optical behavior of a single-junction solar cell with a p-i-n architecture typical in the field [glass/indium-doped tin oxide (ITO)/poly(3,4-ethylenedioxythiophene) (PEDOT)/NBG perovskite/C60/bathocuproine (BCP)/gold (see Figure 1a)].^11,25−28^ To accurately obtain the *J*_sc_ of our solar cell models, we employ refractive indices [*N*(ω) = *n*(ω) + *ik*(ω)] extracted from experimentally achieved materials that have been proven to work in highly efficient devices (see the Supporting Information and Figure S1 for details).^7,10,26,29^ To identify the optimal NBG perovskite, we explore the optical constants of several Pb–Sn perovskites with bandgaps ranging between 1.24 and 1.17 eV reported in the literature.^5,7,10,30,31^*n*(ω) and *k*(ω) spectral values of these materials with high Sn contents (i.e., equal to or more than that of Pb) are represented in panels b and c, respectively, of Figure 1. Given the similarities in nominal perovskite compositions among the depicted examples, the notable distinctions in the refractive index curves are remarkable. These variations underscore the sensitivity of the optical properties within this material family to the intricacies of their fabrication processes, emphasizing the potential implications for the accuracy of calculated results. We illustrate this in Figure S2, where we show significant differences in the electric-field distribution within two solar cells based on very similar perovskite compositions.

**Figure 1:**
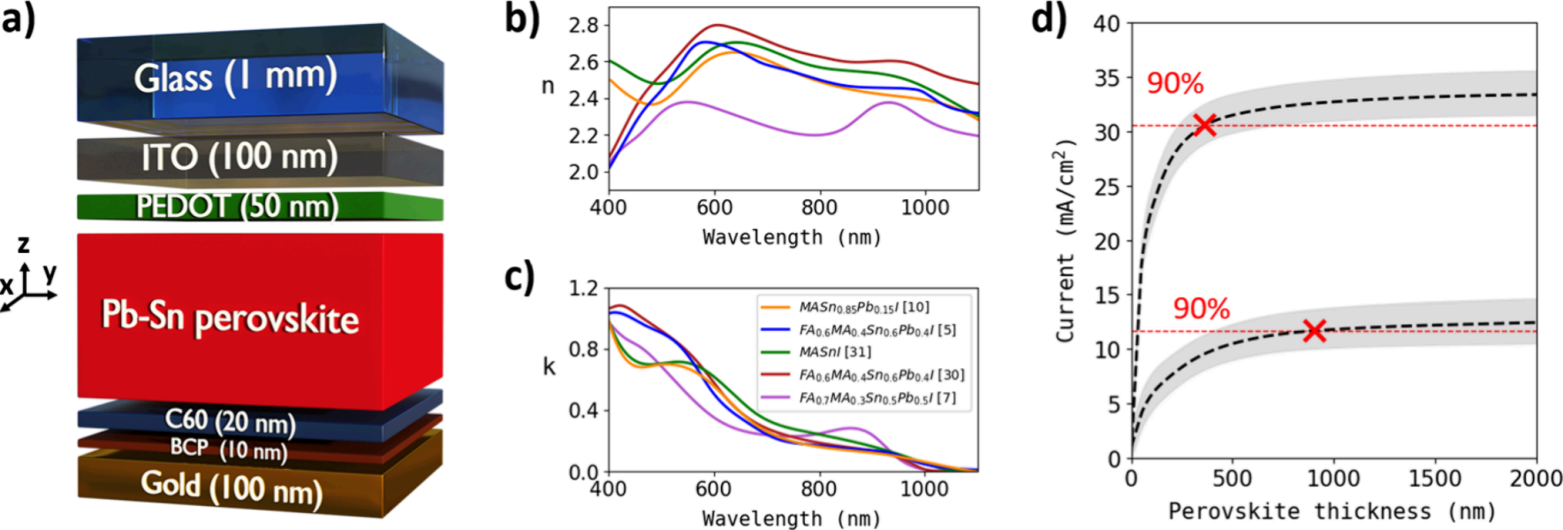
(a) Single-junction solar cell architecture employed for the simulations. (b) Real and (c) imaginary components of the refractive indices for various NGB Pb–Sn perovskites, as reported in the literature. Simulations are performed using these perovskites compositions. (d) Mean calculated photocurrents vs perovskite film thickness. The top halo shows the total current using the entire AM1.5 spectrum, and the bottom one considers only infrared photons reaching the rear subcell in a tandem configuration (i.e., wavelengths from 750 nm onward).

The aforementioned collection of refractive indices is utilized to calculate the potential *J*_sc_ of a Pb–Sn perovskite-based single-junction solar cell with active layer thicknesses of ≤2000 nm (Figure 1d). Dashed curves represent the averaged calculations, while the standard deviation is illustrated by the halo widths. A statistical analysis is conducted for the entire AM1.5 spectrum (top halo) and, specifically, the corresponding NIR range from a wavelength of 750 nm onward (bottom halo). Red dashed lines in Figure 1d represent 90% of the maximum achievable photocurrents for both cases, considering either the entire solar spectrum or only the NIR spectral range. Our calculations indicate that while a thin layer can achieve current saturation by integrating the full AM1.5 solar spectrum, a significantly thicker layer is needed when considering only the NIR range. Specifically, 370 nm of perovskite material is sufficient to saturate the current (*J*_sc_ = 30.6 mA/cm^2^) when considering the full solar spectrum, while 910 nm is required to achieve the equivalent current saturation (in this case, *J*_sc_ = 11.7 mA/cm^2^) when absorbing only NIR photons. This limitation in the ability to absorb longer wavelengths, relevant for tandem solar cells, hampers the practical use of these NBG perovskites in such devices. In addition, the experimental realization of thick perovskite layers presents significant challenges, and their increased thickness leads to larger electrical losses as they approach the diffusion lengths of the charge carriers.^6,32−34^ To strike the trade-off between thickness and efficient charge extraction, we will opt for NBG perovskite layers with a consistent thickness (*h*_*z*_) of 700 nm, which is compatible for experimental realization. We employ the detailed balance to calculate the theoretical *J–V* curves presented in Figure S3,^2,35,36^ finding PCEs ranging between 25.9% and 26.5% for solar cells with an *h*_*z*_ of 700 nm.

With the aim of fully exploiting NIR light, we explore the use of plasmonic NPs randomly distributed within the NBG perovskite layer. We maintain the same p-i-n solar cell configuration as previously described, utilizing a fixed composition of MAPb_0.15_Sn_0.85_I_3_ for the NBG perovskite. This particular composition was chosen because its bandgap is the narrowest of those of the materials mentioned. Additionally, the layer thickness (*h*_*z*_) is set at 700 nm. We carry out simulations in which spherical Ag, gold (Au), and copper (Cu) NPs are randomly distributed and embedded inside the NBG perovskite. We conduct a parameter sweep to find the optimal particle sizes and volume concentrations that maximize the generated photocurrent. The illustration in Figure 2a depicts the simulation domain, essentially representing a unit cell from a macroscopic standpoint. Numerical calculations are conducted with periodic boundary conditions along the *x* and *y* directions, and perfect matched layers (PMLs) are applied along the *z* direction, defining a simulation box size of *L*_*x*_ × *L*_*y*_ × *L*_*z*_. Rigorous numerical checks are implemented to ensure the absence of array effects in the model, avoiding the use of distances between NPs that might induce coupling between their LSPRs. The latter could be possible due to the generally large *k* values of perovskite materials and parasitic absorption in other parts of the cell. The model assumes a random distribution of NPs at midheight within the perovskite film, with variations of both the radius of the sphere (*R*) and the volume filling concentration (VFC) to attain optimized results. Note that VFC represents the ratio between the metallic sphere and NBG perovskite volume, defined as follows:
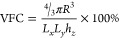
1In Figure 2b, the calculated *J*_sc_ values for various VFCs are depicted as a function of *R* for all of the metals under consideration. This graph specifically highlights the highest-performance cases. Increased concentrations result in a greater displacement of the perovskite material, leading to a reduction in the number of photogenerated carriers, while lower concentrations fail to provide sufficient scattering and electric-field enhancement. The optimal sizes are attributed to synergetic near- and far-field plasmonic effects.^21−23^

**Figure 2:**
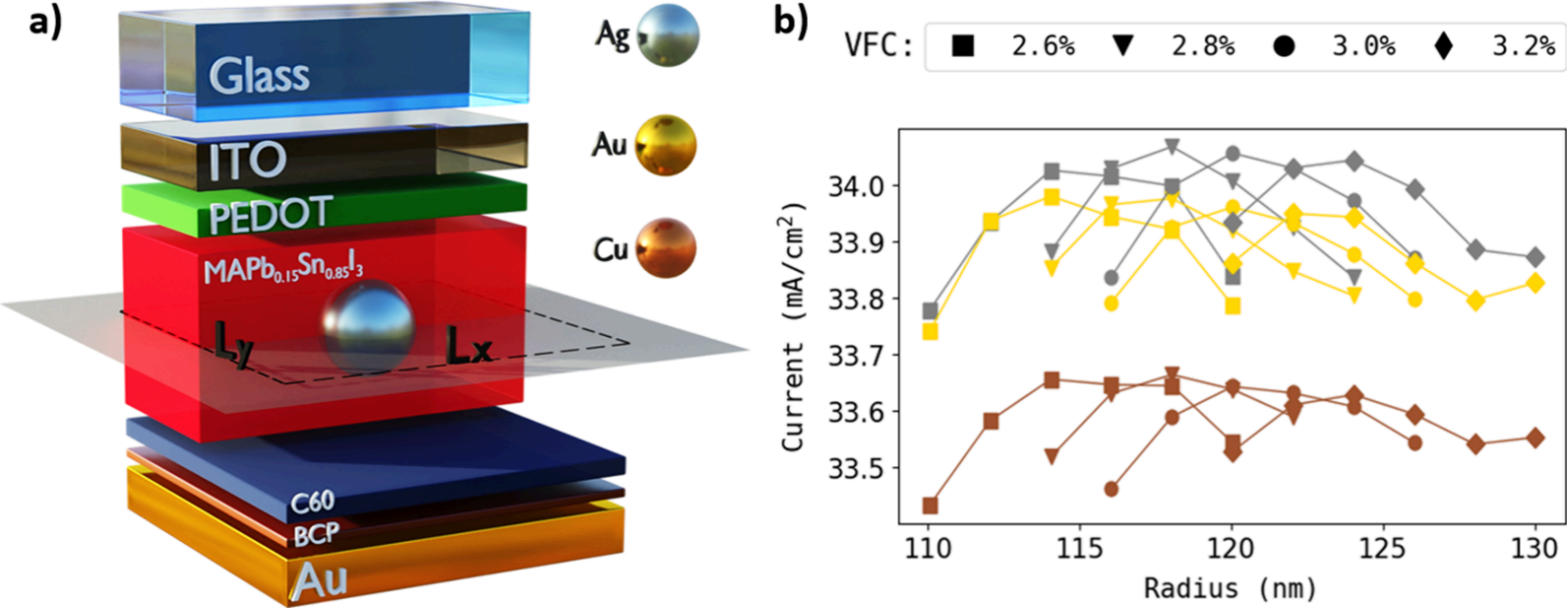
(a) Scheme of the single-junction plasmonic solar cell system employed in the simulations. We consider a glass/ITO (100 nm)/PEDOT (50 nm)/NBG perovskite (700 nm)/C60 (20 nm)/BCP (10 nm)/gold (100 nm) architecture in which metallic spheres (i.e., silver, gold, and copper) are placed in the midplane of the perovskite layer. Both the size and the volume filling concentrations of the particles are varied to produce the optimal performance. (b) Highest calculated currents for different radii and concentrations.

When metallic NPs are taken into account, according to Mie scattering theory,^37^ they commonly demonstrate resonant effective scattering and absorption when exposed to external illumination. The spectral position of these resonant peaks can be adjusted by varying parameters such as *R* (size) or the type of metal used, among others. When these NPs are integrated into our systems to boost the absorption of solar photons, it is crucial for these resonant peaks to align with spectral ranges characterized by intense solar irradiance. As one can see in Figure 2b, the optimal sphere size that maximizes *J*_sc_ in the NBG-based single-junction solar cell corresponds to an *R* of 118 nm, for the three metals considered. In good agreement with previous results focused on pure Pb single-junction devices,^21,22^ Ag NPs afford the best results. The high absorption characteristics of perovskite materials inhibit the application of scattering Mie theory, which is suitable fo only low-absorbing external media.^37−39^Figure S4 showcases the Mie efficiencies of spherical NPs, which are submerged in a non-absorbing medium (*k(ω)* = 0) with the *n*(ω) of our perovskite material. By examining the effective absorption cross sections of these metals, we observe that Cu and Au exhibit higher values, but it becomes evident that Ag offers superior effectiveness in plasmonic approaches due to its ability to minimize parasitic absorption. The results depicted in Figure 2b demonstrate a remarkable improvement by establishing a potential current *J*_sc,record_ of 34.05 mA/cm^2^ in single-junction solar cells based on Pb–Sn perovskite materials. This outstanding outcome is achieved with Ag NPs (*R* = 118 nm; VFC = 2.8%), yielding a substantial increase of 1.1 mA/cm^2^ in the extracted photocurrent compared to that of the reference cell (*J*_sc,ref_ = 32.94 mA/cm^2^), translating to a nearly 3.5% enhancement of the extracted photocurrent through our photonics design. In addition to the achieved record system, a considerable range of *R* and VFC values results in a notable current enhancement. This indicates that the system is not highly sensitive to variations in the optimal parameters, making the designed scheme experimentally feasible. Figure S5 shows the same calculations but considers only low-energy photons, namely those with λ values >750 nm, corresponding to the spectral range of interest for tandem devices. As one can see, the shape of this figure is nearly identical to that in Figure 2b, revealing that the performance improvement primarily arises from NIR absorption enhancement as hypothesized.

Figure 3a shows the comparison between the total absorptance spectra of a single-junction reference cell (blue line) and the optimal plasmonic single-junction cell containing Ag NPs with an R of 118 at a VFC of 2.8% (orange line). The plasmonic cell exhibits enhanced absorption in the NIR region. However, to calculate the current generated by the solar cell, it is crucial to determine the productive absorptance, considering only photons absorbed within the perovskite volume and excluding parasitic absorption in the metallic sphere and other layers of the solar device. The productive absorption is determined by integrating the differential absorption per unit volume, *δA*, in the perovskite volume. Further details of these calculations are provided in the Supporting Information. Additionally, the figure includes the ratio of the productive absorptances of both cells (represented by the green line), revealing a plasmon resonance in the NIR range. Panels b and c of Figure 3 illustrate *δA* over a cross-sectioned plane (*y* = 0) of the NBG perovskite layer at two specific wavelengths: 916 nm (top panels) and 1038 nm (bottom panels), both of which exhibit significantly improved absorptance compared to the reference case in Figure 3a. The left panels depict systems with incorporated plasmonic NPs, while the right panels show devices without them. In the plasmonic and reference cases, Fabry–Perot resonances are observed due to thin film interference effects. However, the plasmonic cell additionally benefits from high-order LSPRs, resulting from the large particle size and high refractive index of the external perovskite medium. The coupling of LSPRs with interference effects amplifies the electric field around the NPs, maximizing the perovskite absorption at the resonant wavelengths. Hence, to maximize the photocurrent, such coupling must be achieved at wavelengths for which the solar spectrum (i.e., AM1.5) is more intense. Additionally, it is worth noting that, as the extinction coefficient decreases (see Figure 1c), particularly at λ =1038 nm, near-field effects become more prominent because the electric-field intensity reaching the NP is higher. Therefore, the strongest near-field effects are observed in the spectral region where the perovskite material absorbs less efficiently, even doubling the absorptance near the absorption edge, as shown in Figure 3a.

**Figure 3:**
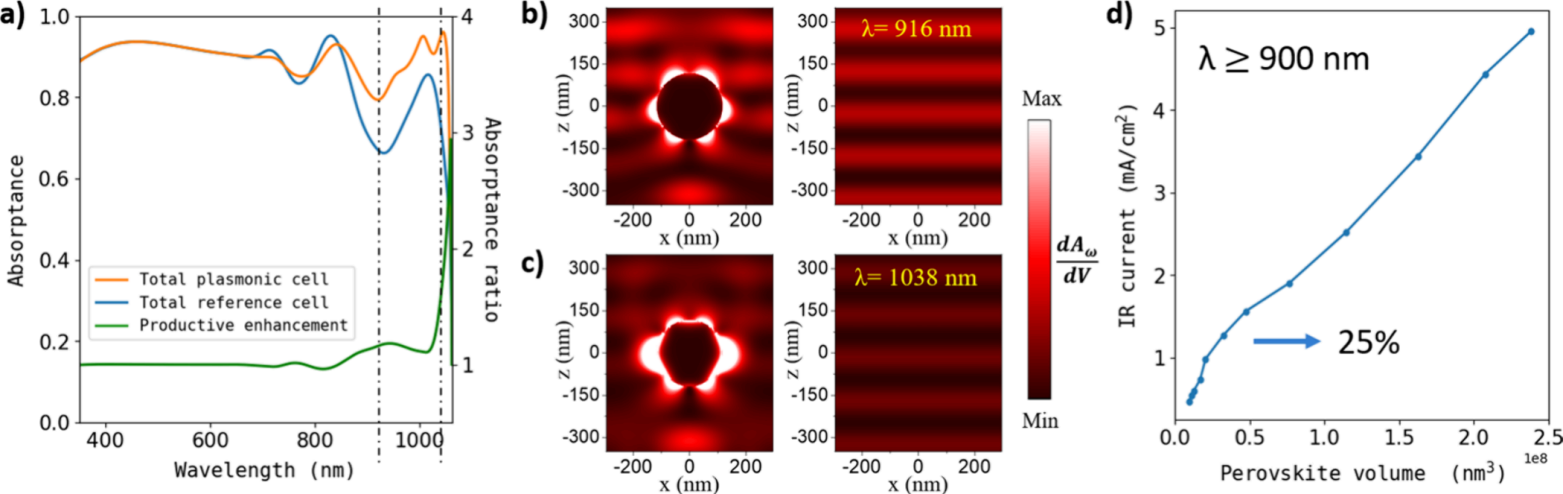
Enhancement of the performance of a single-junction solar cell with a glass/ITO (100 nm)/PEDOT (50 nm)/NBG perovskite (700 nm)/C60 (20 nm)/BCP (10 nm)/gold (100 nm) architecture through plasmonic effects. (a) Total absorptance spectrum of the reference system (blue) compared with that of the record system (orange) incorporating Ag NPs with an *R* of 118 nm and a VFC of 2.8%. The ratio of the productive absorptances (i.e., those considering only the photons absorbed by the perovskite material) is also displayed (green), demonstrating a plasmon resonance and a considerable absorption enhancement at λ >750 nm. Differential absorption per unit of volume profiles depicted at (b) λ = 916 and (c) λ = 1038 nm. Reference (right) and record (left) systems are compared. The images show an absorption enhancement where the plasmonically induced multipolar resonance couples the interferential patterns. (d) Current generation calculated over increasing concentric volumes around the Ag sphere. Currents are calculated using only λ >900 nm photons, a regime in which the plasmonic enhancement is more intense. The observed change in the curve slope is attributed to the vanishing near-filed enhancement. Hence, it is estimated that 25% of the enhancement is due to near-field effects, in contrast to far-field (scattering) gain.

We now proceed to investigate both near- and far-field (scattering) contributions to the absorption enhancement in our record system. To achieve this, we conducted simulations in which we calculate the photogenerated current in the NBG perovskite across expanding integration cubic volumes concentric to the NP (see Figure 3d). This approach allows us to distinguish near-field effects from far-field effects. Here, we consider only photons with λ >900 nm, the spectral range in which plasmonic enhancement plays a major role. The curve shows an abrupt change in the slope around volumes of 3 × 10^7^ nm^3^, corresponding to approximate distances of ∼ 40 nm from the surface of the sphere, revealing the extent of the near-field influence. It is thus estimated that 25% of the current generation is due to near-field effects in this spectral region of interest, in agreement with previously reported effects with Ag nanocubes inside Pb-based perovskite films.^22^ This result highlights the interest in introducing metal NPs directly embedded within the absorbing layer, providing an additional near-field contribution to scattering effects that would otherwise be wasted.

Now that the NBG perovskite single cell has been optimized, we move on to the tandem configuration. The simulated system consists of a glass/ITO/WBG perovskite/C60/ALD-SnO_2_/PEDOT/NBG perovskite/C60/BCP/Au stack (see Figure S6), in which the WBG perovskite composition is FA_0.7_Cs_0.3_Pb(I_0.7_Br_0.3_)_3._ According to the detailed balance model in tandem devices, this WBG material possesses a bandgap that, when combined with the selected NBG perovskite in a tandem device, favors optimal harvesting of the solar spectrum.^40^ For this scenario, widely employed experimentally feasible geometrical parameters will also be considered. The development of self-assembled monolayers (SAMs) has allowed the use of extremely thin layers that minimize parasitic absorption in perovskite solar cells.^8,9,25^ For this reason, the thin MeO-2PACz film acting as a hole selective layer at the front subcell will not affect the electric-field distribution and therefore will not be considered in our calculations. The refractive indices used for the tandem solar cell simulations are taken from the literature.^7,10,26,29,40^

To advance the tandem solar cell with embedded plasmonic NPs, a new optimization is required to determine the optimum thickness of the front WBG perovskite layer. Finding this optimal thickness is crucial, as it impacts the interference pattern and its coupling to LSPRs, which may differ with the presence of additional layers. Nevertheless, for this optimization, we will conduct a geometrical parameter sweep near the previous record case and exclusively consider the use of Ag NPs. In these simulations, with the NBG layer thickness fixed at 700 nm, the thickness of the WBG layer is adjusted until the calculated currents in the front and rear subcells match, within the range of 340–350 nm, thereby maximizing the total device current. NP optimization is represented in Figure 4a, where matched currents at different VFC and *R* values are shown. It is found that spherical Ag NPs with an *R* of 120 nm and a VFC of 3.1% embedded in a 350 nm WBG perovskite layer give rise to a *J_sc,record_*of 16.37 mA/cm^2^, corresponding to a potential overall PCE of 33.34%, in comparison to that of the reference tandem (31.33%). Remarkably, the particle size and concentration closely align with the optimal values attained for single-junction plasmonic solar cells (*R* = 118 nm, and VFC = 2.8%). These values fall within the experimental error range observed during the fabrication of these devices. This suggests that optimizing the single-junction plasmonic solar cell would effectively result in high-performance devices within the parameter range considered in this study, while considerably reducing computational time and the need for extensive parameter sweeps.

**Figure 4:**
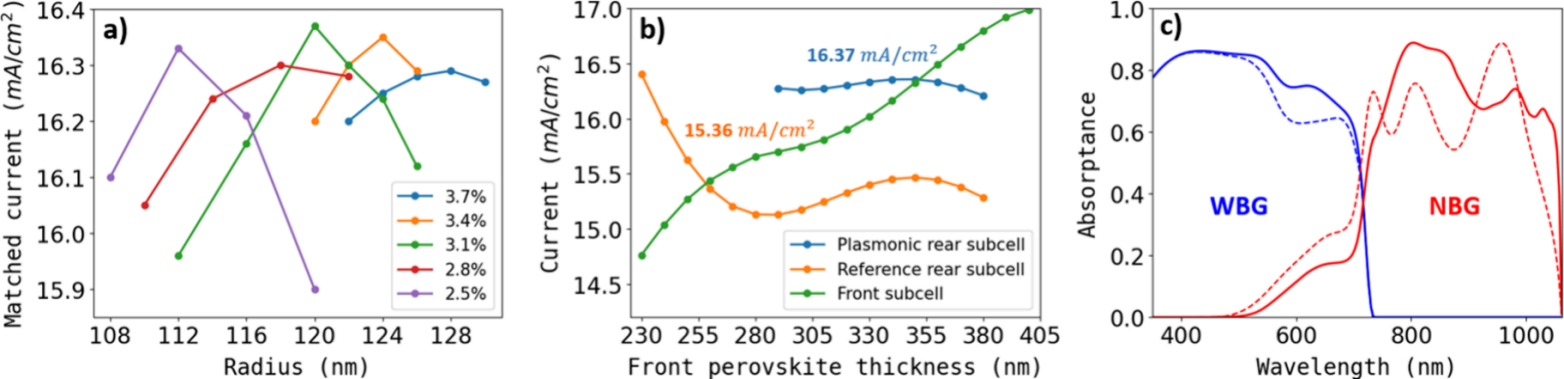
(a) Optimization of the sizes and volume filling concentrations of Ag NPs embedded in 700 nm thick NBG perovskites for tandem devices, with a variable thickness of the WBG layer between 340 and 350 nm. (b) Record plasmonic tandem system (*R* = 120 nm, and VFC = 3.1%) compared to a reference tandem device. The photogenerated currents in each subcell are plotted against the thickness of the WBG perovskite, showing the fulfilment of current matching conditions. (c) Productive absorptance of both reference (dashed lines) and record (solid lines) tandem devices. WBG layer (blue lines) and NBG layer (red lines) absorptance contributions are also depicted.

In Figure 4b, we compare the current evolution in the rear and front subcells with varying WBG perovskite film thicknesses. The reference tandem cell and the plasmonic tandem cell with optimal NPs in Figure 4a are showcased. Notably, the reference tandem requires a 260 nm WBG layer for current matching (*J*_sc,ref_ = 15.36 mA/cm^2^), while the record tandem device needs 350 nm, providing the maximum *J*_sc__,record_ of 16.37 mA/cm^2^. In Figure 4c, we compare the absorptance spectra of these cells, revealing significant enhancement, particularly in the infrared region. However, considering the experimental realization of these models, the direct introduction of Ag NPs into perovskite materials can lead to chemical instability. To address this limitation, we propose exploring alternative NP geometries, such as core–shell nanospheres.^41−43^ Simulations have also been conducted with silica-coated Ag NPs (Figure S7). The results indicate a reduction in the calculated current as the silica shell thickness increases, specifically, 0.26 mA/cm^2^ for a 10 nm shell. Consequently, further optimization of the system configuration is necessary if we modify the NP structure. Furthermore, as absorption enhancement stems from plasmon coupling with the interference pattern, the position of the NPs along the *z*-axis can influence the calculated efficiency. This was previously examined in pure Pb perovskite thin films.^23^ In line with this, results presented in the Supporting Information showcase the potential impact of the NP distribution along the *z*-axis on the efficiency of a real device. Specifically, we investigate scenarios in which the NPs are located along the *z*-axis, either at the midplane or out of it (Figure S8). While our focus is primarily on optical aspects, plasmonic particles have been proposed to enhance electrical properties. Indeed, previous studies have highlighted the potential of hot electron transfer and plasmonically induced energy transfer, altering carrier dynamics and suppressing nonradiative recombination.^44−46^

In summary, our study reveals a substantial improvement in the calculated photocurrent for both single- and double-junction all-perovskite solar cells by finely tuning the cell architecture and leveraging plasmonic effects. The optimized Pb–Sn perovskite layer in the single-junction model achieves a remarkable 3.5% current enhancement, resulting in an estimated PCE of 27.4%, utilizing Ag NPs with a radius of 118 nm and a 2.8% volume concentration directly embedded in the NBG material. In a tandem configuration, our approach affords a PCE of 33.37%, marking a >2% absolute enhancement and showcasing the potential for unprecedented efficiencies in perovskite–perovskite tandem devices. This performance boost is realized through the synergy of high-order localized surface plasmon resonances with interference effects in the thin Pb–Sn perovskite layer, ensuring efficient light absorption. Our strategy allows for a remarkable reduction in the thickness of Pb–Sn perovskite solar cells, offering a pathway for enhanced charge extraction while diminishing the weight and the use of toxic lead.

## Supporting Information Available

More details of the simulation methods and optical constants of the materials employed (PDF)
