# Supplementary material for: Synergetic Near- and Far-Field Plasmonic Effects for Optimal All-Perovskite Tandem Solar Cells with Maximized Infrared Absorption
Source: J Phys Chem Lett. 2024 Feb 29;15(9):2632–8. doi: 10.1021/acs.jpclett.4c00194 (PMC10926158; doi:10.1021/acs.jpclett.4c00194)
Supplement: Supplementary file 1 — jz4c00194_si_001.pdf [file jz4c00194_si_001.pdf]

Supporting information

Synergetic Near- and Far-Field Plasmonic Effects for Optimal All-Perovskite Tandem Solar Cells with Maximized Infrared Absorption

Jaime Bueno[1], Sol Carretero Palacios[,1], Miguel Anaya*[,2]*

[1]Instituto de Ciencia de Materiales de Madrid, ICMM-CSIC, C/Sor Juana Inés de la Cruz, 3, 28049 Madrid, Spain.

[2]Departamento de Física de la Materia Condensada, Instituto de Ciencia de Materiales de Sevilla, Universidad de Sevilla-CSIC, Av. Reina Mercedes SN, Sevilla, 41012, Spain.

Corresponding Author

*Correspondence and requests for materials should be addressed to S. C. P. (sol.carretero@csic.es) and M. A. (anaya@us.es)

A. Single-junction cell simulation details

Real ($n(\lambda)$) and imaginary ($k(\lambda)$) parts of the refractive indices extracted from the literature and used as input in the simulations for the device stack shown in Figure 1a, are represented in **Figures S1a y S1b**, respectively. Glass was modelled as a dielectric material with a constant refractive index of 1.5.

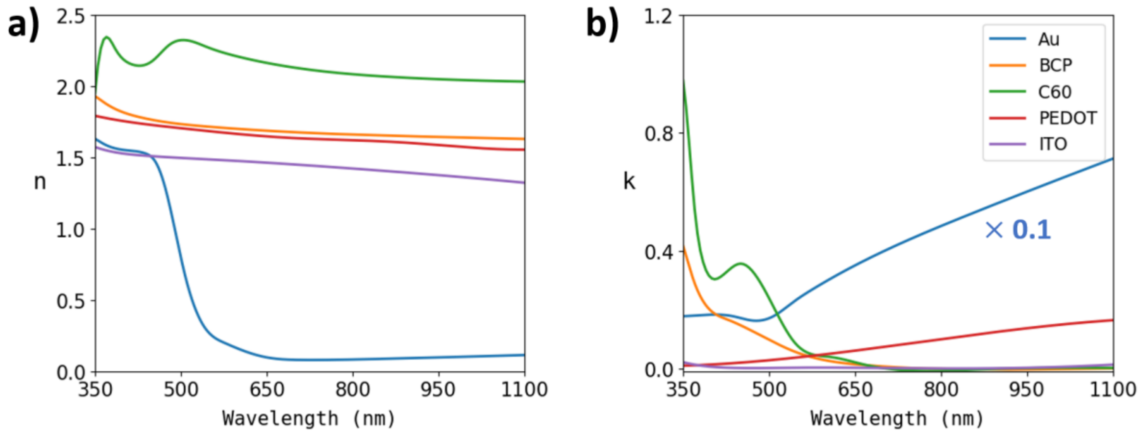

Figure S1. a) *Single-junction solar cell architecture, and corresponding material thicknesses used to perform optical simulations. All employed materials are characterized by their refractive indices, both real ($n(\lambda)$)* ***(b)*** *and imaginary ($k(\lambda)$)* ***(c)*** *parts. This information is taken from the literature.*[1–4]

Numerical calculations were performed using Ansys Lumerical FDTD software.[5] The device stack and corresponding material thicknesses used are displayed in Figure 1a. For the simulations, periodic boundary conditions were used along *x* and *y* axis, and perfect matching layers (PMLs) were used at *z* axis. The shown device stack was illuminated by a normalized plane wave from above. Due to computational requirements, the light source and PML upper limit were established inside the glass substrate, since in real devices it is about one millimetre thick, far exceeding the order of magnitude of the simulation. To compensate for this, the plane wave intensity was normalized to 0.96 to model air-glass interface reflection.

During the propagation of the wave through the solar cell architecture, the electric field (E) within the perovskite layer is recorded, and the absorptance within the whole system is calculated as follows:

$$A_\omega = \iiint \omega \varepsilon_0 |E_\omega(x,y,z)|^2 n_\omega k_\omega \, dxdydz$$

where ε_0 is the vacuum permittivity. This allows discriminating parasitic and productive absorption, by integrating the number of absorbed photons at a certain frequency ω over the different material spatial regions. Then, productive absorptance in perovskite layers is multiplied by the photon flux coming from the Sun that reaches Earth surface, i.e., AM1.5 spectrum. Integrating over the spectral range of interest, the total number of absorbed photons can be calculated. Thus, the ideal photogenerated current density, assuming that each absorbed photon produces an electron that contributes to the photocurrent, is calculated as follows:[6]

$$J_{ideal} = q \int A_\omega \, AM1.5_\omega \, d\omega$$

being q the electron charge.

Figures S2a and S2b depict the electric field distribution within the perovskite film in two single-junction solar cells with distinct compositions ($MAPb_{0.15}Sn_{0.85}I_3$ in panel a, and $FA_{0.6}MA_{0.4}Sn_{0.6}Pb_{0.4}I_3$ in panel b). The outcomes underscore a pronounced reliance on the refractive index of the materials.

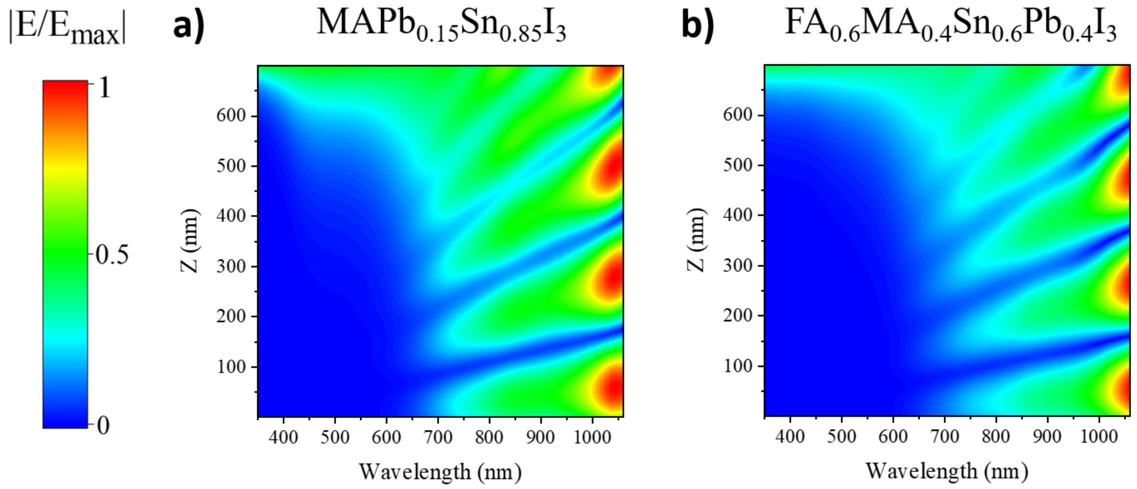

***Figure S**2. Electric field profile inside the 700 nm thick Pb-Sn perovskite layer of the single-junction solar cell model employed for the simulations. Two perovskite compositions **a)** $MAPb_{0.15}Sn_{0.85}I_3$ and **b)** $FA_{0.6}MA_{0.4}Sn_{0.6}Pb_{0.4}I_3$ are ilustrated with the aim of showing the strong dependence of the calculations with the refractive indices of such similar materials.*

Utilising the calculated ideal currents from Figure 1d in the main manuscript for 700 nm thick perovskite layers, we employ detailed balance principles to derive J-V curves.[6–8] These curves are computed considering all incident photons (AM1.5 spectrum) and, separately, focusing on infrared (IR) photons with wavelengths exceeding 750 nm, the spectral range these perovskites absorb in a tandem configuration. Notably, the J-V curves for the IR spectral region exhibit minimal changes in open circuit voltages compared to those considering the entire AM1.5 spectrum. This observation underscores the potential for achieving high efficiencies in tandem devices through enhanced absorption in the near-infrared (NIR) region.

Figure S3 shows the J-V curves for a 700 nm Pb-Sn perovskite layer of diverse composition in a single junction solar cell, discriminating the current contribution of NIR photons (i.e., those with $\lambda > 750$ nm).

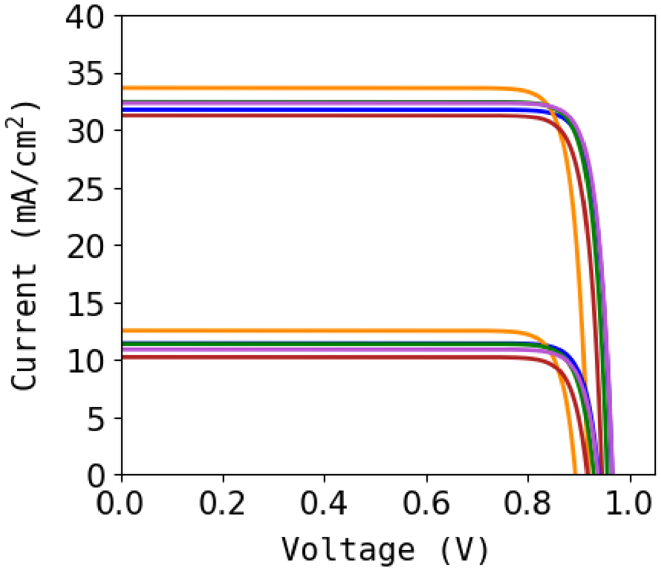

Figure S3. *J-V curves depict the performance of single-junction solar cells with a 700 nm thick Pb-Sn perovskite layer, showcasing various compositions. The colour code aligns with Figure 1b and Figure 1c in the main manuscript. Calculated based on the ideal photocurrents from simulations using the reported single-junction model and following detailed balance principles, the top curves represent ideal currents considering all incident photons (AM1.5), while the bottom panels focus on photons with* $\lambda > 750$ *nm.*

B. Plasmonic cell optimisation

Plasmonic nanoparticles (NPs) are embedded in the narrow bandgap (NBG) perovskite layer to benefit from far-field and near-field plasmonic effects. **Figure S4** illustrates the scattering and absorption efficiencies of spherical NPs made of Silver, Gold, and Copper. The refractive indices of the three materials were taken from well-established models, namely those of Johnson and Christy, Babar and Weaver, and Palik, respectively. Due to the limitations of Mie theory in highly absorbing external media,[9] we conducted our calculations in a non-absorbing ($k = 0$) external Pb-Sn perovskite medium, while preserving the real part of the optical constants. The scattering and absorption cross-sections were obtained using the Mie scattering theory,[10,11] and these values were normalized to the sphere cross-section (i.e., $\pi \cdot R^2$) to determine the efficiencies. Upon analysing the results, it is evident that silver NPs (**Figure S4a**) exhibit superior plasmonic qualities for our specific application of maximising light absorption in the embedding Pb-Sn medium. This conclusion is drawn from the observation of higher scattering cross-sections and lower absorption cross-sections in the spectral region of interest (λ > 750 nm) compared to Gold (**Figure S4b**) and Copper (**Figure S4c**) NPs.

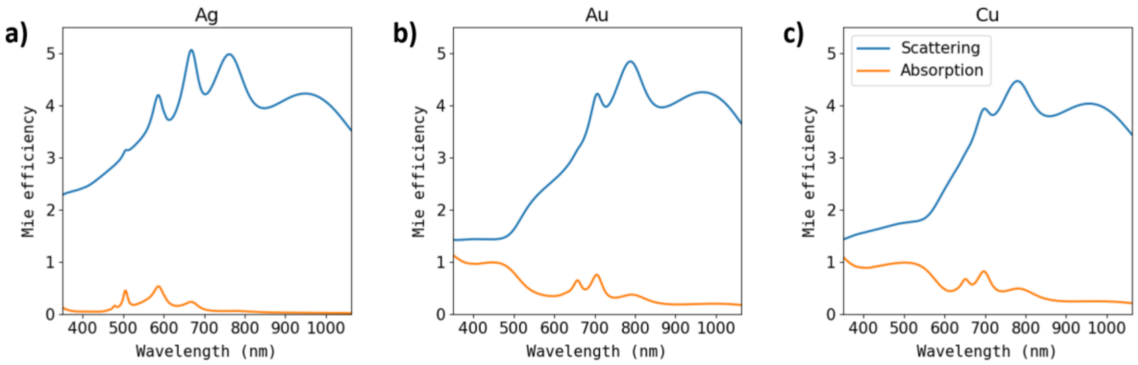

Figure S4. *Scattering (in blue lines) and absorption (in orange lines) cross-section efficiencies obtained with Mie scattering theory for Silver (**a**), Gold (**b**) and Copper (**c**) spheres of 118 nm of radius embedded in a non-absorbing ($k = 0$) Pb-Sn perovskite medium.*

Figure S4 depicts calculations using spherical NPs with a radius of 118 nm. As demonstrated in the main text (see Figure 2b), these NPs exhibit optimal performance

when included with a 2.8% volume concentration in the absorbing layer of a Pb-Sn-based single-junction solar cell. Moving in the same vein, **Figure S5** showcases similar optimizations as presented in Figure 2b from the main manuscript, focusing exclusively on the current generated by NIR photons, particularly those with a wavelength exceeding 750 nm. Notably, the optimal NP sizes and concentrations remain consistent, resulting in a record $J_{sc,record}^{\lambda>750\,nm}$ = 13.72 mA/cm^2 for Ag NPs. This represents a substantial relative increase of over 9% compared to a reference cell lacking NPs ($J_{sc,ref}^{\lambda>750\,nm}$ = 12.56 mA/cm^2). Furthermore, the resulting curves mirror those in Figure 2b, indicating that the performance improvement primarily stems from NIR absorption enhancement. This behaviour can be elucidated in terms of the Mie efficiencies discussed above.

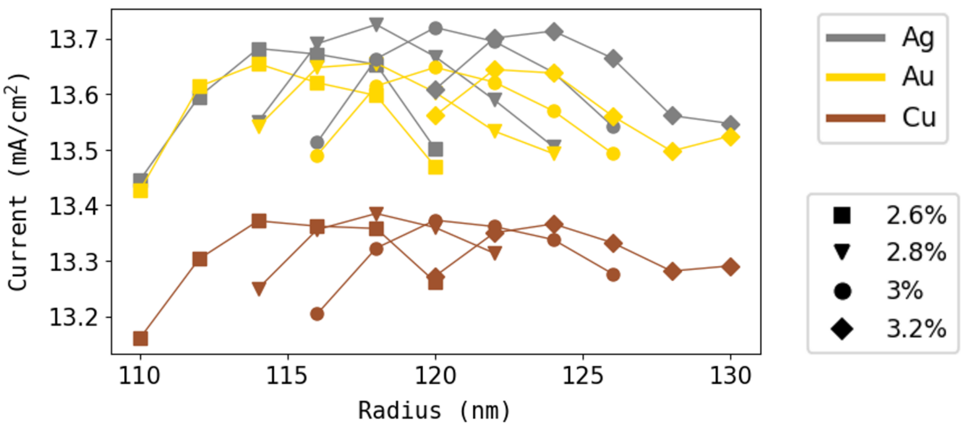

Figure S5. *Current produced by NIR photons (λ >750 nm) in a single-junction solar cell with embedded NPs. Metallic spheres (i.e., Silver, Gold and Copper) of different radius are included inside the perovskite layer with different volume filling concentrations.*

C. Tandem solar cell simulation details

Simulations for tandem configurations based on a double junction all-perovskite solar cell are performed following the same methods as for the single-junction solar cell. As it can be seen in **Figure S6a**, additional layers are added in the multijunction solar architecture, including the WBG perovskite layer, with a $FA_{0.7}Cs_{0.3}Pb(I_{0.7}Br_{0.3})_3$ composition. Real and imaginary parts of the refractive indices of the additional materials are shown in **Figure S6b**.

Absorption at each perovskite layer is calculated separately to discern between each current density contribution. NBG layer thickness in the rear subcell is fixed to 700 nm, while the WBG layer thickness in the front subcell is swept from 300 nm to 380 nm, in which the relative positions of all layers are reconfigured according to the evaluated WBG thickness, to attain and maximize current matching conditions, i.e., when front and rear subcells generate the same current.

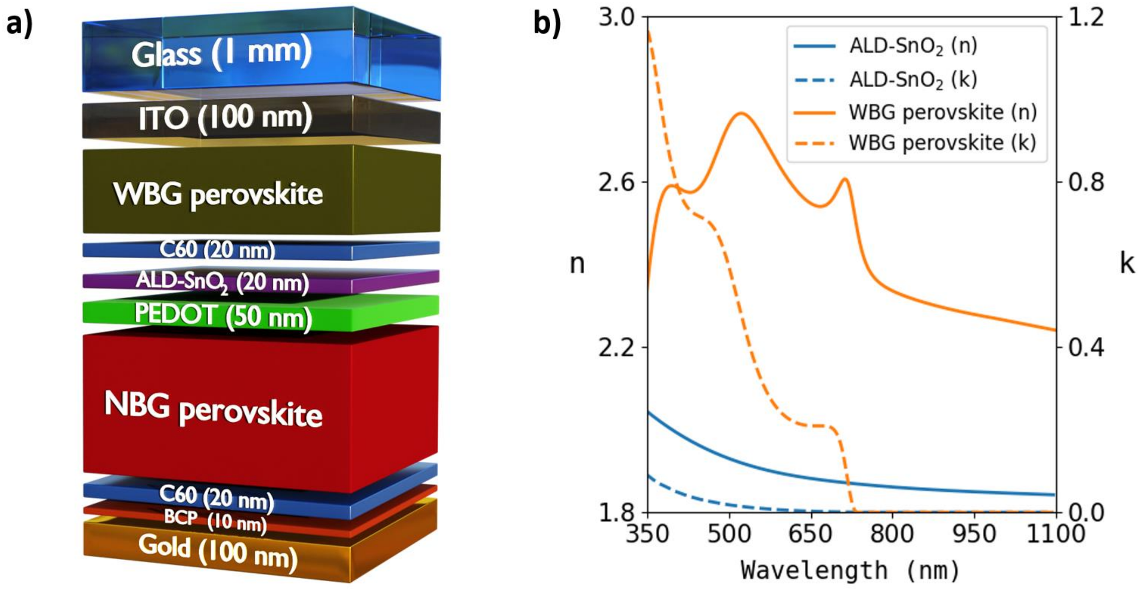

***Figure S6. a)** Scheme of the architecture of an all-perovskite tandem solar cell used to perform optical simulations. **b)** Complex refractive indices of aditional materials employed in tandem cell simulations extracted from* [1,12].

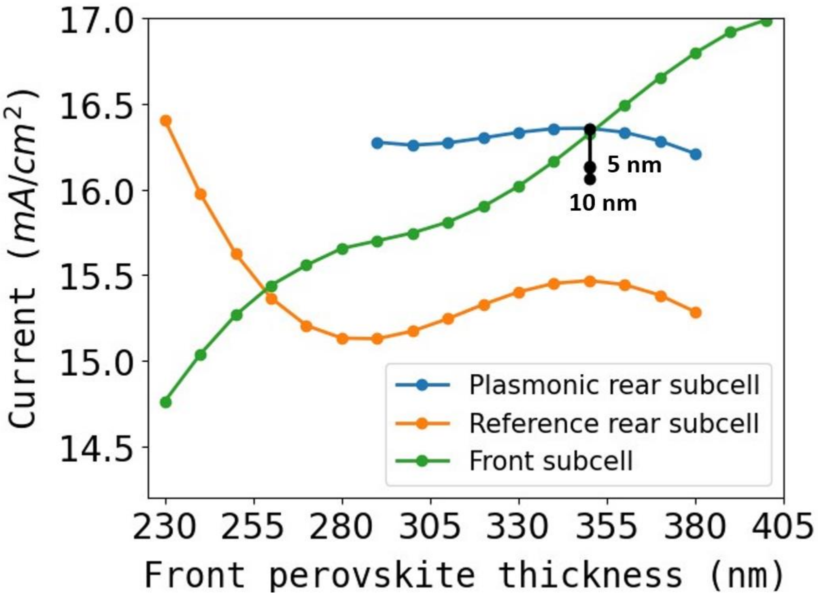

Figure S7. *Record plasmonic tandem system (R=120 nm, VFC=3.1%) is compared to a reference tandem device. The photogenerated currents in each subcell are plotted against the thickness of the WBG, showing the current matching conditions fulfilment. For the specific case of 350 nm thick WBG, close to the current matching condition, the current generated in the rear subcell has been calculated when coating the NP with silica shells of 5 and 10 nm.*

D. <u>Details of particle location effects along the z-axis</u>

The coupling between thin film interference pattern and the plasmonic effects coming from the NP could be affected by any structural change in the device model, i.e., the thicknesses of the layers or the position of the NP. Note that, for this study, due to the extensive computational requirements, we have always considered the NP fixed at mid-height inside the NBG layer, relying on the presence of the strongest synergistic near- and far-field plasmonic effects.[13] Nevertheless, in a real device, a complete random distribution of the NPs all along the vertical axis is more likely. Thus, here we quantify the impact of such NP distribution in our optimized model. Additional calculations where the NP is embedded at heights z = -175 nm and z = 175 nm were performed. Absorption profiles of these systems, along with the z = 0 nm case, are shown in Figure S8. The selected wavelength, λ= 858 nm, corresponds to the spectral value at which the maximum enhancement is observed compared to the non-plasmonic reference cell, at z = 0 nm. Averaging these three contributions, the generated current is reduced to J_{sc} = 16 mA/cm^2. Note that this result was obtained using the optimal NPs (R = 120 nm and VFC = 3.1 %)

and WBG layer thickness already calculated, resulting in lower plasmonic induced enhancements, and thus, also in the subcell current mismatching. Taking this into account, further optimisations including averaged calculations with NPs along all vertical axis should be done in order to maximize the PCE.

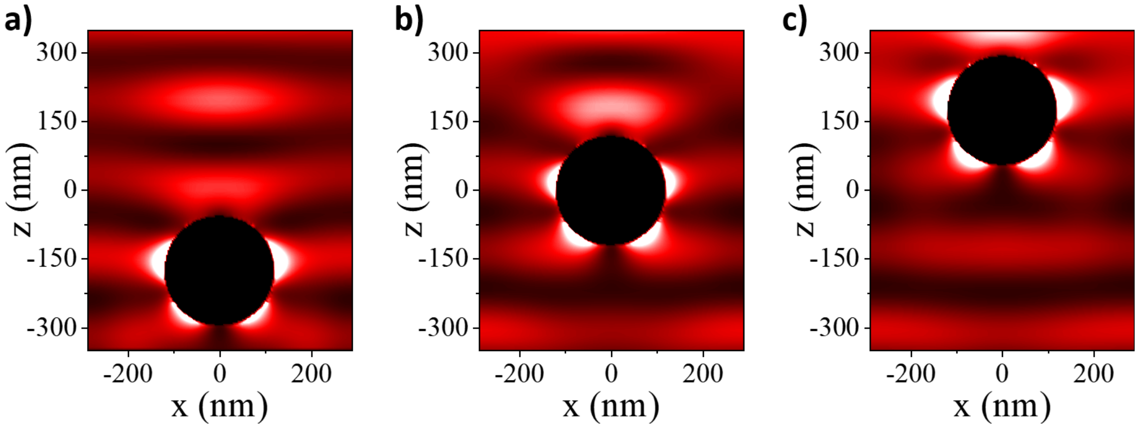

Figure S8. *Differential absorption per unit of volume in the NBG perovskite layer of a tandem device. The NP is located at* ***a)*** *z = -175 nm,* ***b)*** *z = 0 nm and* ***c)*** *z = 175 nm. The color scale follows that of the same manuscript.*

REFERENCES

(1) Xiao, K.; Lin, R.; Han, Q.; Hou, Y.; Qin, Z.; Nguyen, H. T.; Wen, J.; Wei, M.; Yeddu, V.; Saidaminov, M. I.; Gao, Y.; Luo, X.; Wang, Y.; Gao, H.; Zhang, C.; Xu, J.; Zhu, J.; Sargent, E. H.; Tan, H. All-Perovskite Tandem Solar Cells with 24.2% Certified Efficiency and Area over 1 Cm2 Using Surface-Anchoring Zwitterionic Antioxidant. *Nat Energy* **2020**, *5* (11), 870–880. https://doi.org/10.1038/s41560-020-00705-5.

(2) Hossain, M. I.; Hasan, A. K. M.; Qarony, W.; Shahiduzzaman, Md.; Islam, M. A.; Ishikawa, Y.; Uraoka, Y.; Amin, N.; Knipp, D.; Akhtaruzzaman, Md.; Tsang, Y. H. Electrical and Optical Properties of Nickel-Oxide Films for Efficient Perovskite Solar Cells. *Small Methods* **2020**, *4* (9), 2000454. https://doi.org/10.1002/smtd.202000454.

(3) Anaya, M.; Correa-Baena, J. P.; Lozano, G.; Saliba, M.; Anguita, P.; Roose, B.; Abate, A.; Steiner, U.; Grätzel, M.; Calvo, M. E.; Hagfeldt, A.; Míguez, H. Optical Analysis of $CH_3NH_3Sn_xPb_{1-x}I_3$ Absorbers: A Roadmap for Perovskite-on-Perovskite Tandem Solar Cells. *J. Mater. Chem. A* **2016**, *4* (29), 11214–11221. https://doi.org/10.1039/C6TA04840D.

(4) Baum, M.; Alexeev, I.; Latzel, M.; Christiansen, S. H.; Schmidt, M. Determination of the Effective Refractive Index of Nanoparticulate ITO Layers. *Opt. Express* **2013**, *21* (19), 22754. https://doi.org/10.1364/OE.21.022754.

(5) Lumerical Inc.

(6) Rühle, S. Tabulated Values of the Shockley–Queisser Limit for Single Junction Solar Cells. *Solar Energy* **2016**, *130*, 139–147. https://doi.org/10.1016/j.solener.2016.02.015.
(7) Shockley, W.; Queisser, H. J. Detailed Balance Limit of Efficiency of *P-n* Junction Solar Cells. *Journal of Applied Physics* **1961**, *32* (3), 510–519. https://doi.org/10.1063/1.1736034.
(8) Kirchartz, T.; Rau, U. Detailed Balance and Reciprocity in Solar Cells. *phys. stat. sol. (a)* **2008**, *205* (12), 2737–2751. https://doi.org/10.1002/pssa.200880458.
(9) Sudiarta, I. W.; Chylek, P. Mie-Scattering Formalism for Spherical Particles Embedded in an Absorbing Medium. *J. Opt. Soc. Am. A, JOSAA* **2001**, *18* (6), 1275–1278. https://doi.org/10.1364/JOSAA.18.001275.
(10) Mie, G. Beiträge Zur Optik Trüber Medien, Speziell Kolloidaler Metallösungen. *Annalen der Physik* **1908**, *330* (3), 377–445. https://doi.org/10.1002/andp.19083300302.
(11) Bohren, C. F.; Huffman, D. R. *Absorption and Scattering of Light by Small Particles*; Wiley-VCH: Weinheim, 2004.
(12) Bowman, A. R.; Lang, F.; Chiang, Y.-H.; Jiménez-Solano, A.; Frohna, K.; Eperon, G. E.; Ruggeri, E.; Abdi-Jalebi, M.; Anaya, M.; Lotsch, B. V.; Stranks, S. D. Relaxed Current Matching Requirements in Highly Luminescent Perovskite Tandem Solar Cells and Their Fundamental Efficiency Limits. *ACS Energy Lett.* **2021**, *6* (2), 612–620. https://doi.org/10.1021/acsenergylett.0c02481.
(13) Carretero-Palacios, S.; Calvo, M. E.; Míguez, H. Absorption Enhancement in Organic–Inorganic Halide Perovskite Films with Embedded Plasmonic Gold Nanoparticles. *J. Phys. Chem. C* **2015**, *119* (32), 18635–18640. https://doi.org/10.1021/acs.jpcc.5b06473.
